# External validation of serum hCG cutoff levels for prediction of resistance to single-agent chemotherapy in patients with persistent trophoblastic disease

**DOI:** 10.1038/sj.bjc.6604849

**Published:** 2009-03-17

**Authors:** L G Kerkmeijer, C M Thomas, R Harvey, F C Sweep, H Mitchell, L F Massuger, M J Seckl

**Affiliations:** 1 Department of Chemical Endocrinology, Radboud University Nijmegen Medical Centre, PO Box 9101, Nijmegen 6500 HB, The Netherlands; 2 Department of Obstetrics and Gynaecology, Radboud University Nijmegen Medical Centre, PO Box 9101, Nijmegen 6500 HB, The Netherlands; 3 Department of Medical Oncology, Charing Cross and Hammersmith Campuses Imperial College, London W6 8RF, UK

**Keywords:** combination chemotherapy, drug resistance, methotrexate, persistent trophoblastic disease, single-agent chemotherapy

## Abstract

Van Trommel *et al* have previously shown that serum human chorionic gonadotropin (hCG) cutoff levels can provide early prediction of resistance to first-line methotrexate (MTX) in patients with persistent trophoblastic disease (PTD). In this study, we validate this approach of prediction of resistance to single-agent chemotherapy in an independent and larger cohort of PTD patients using a different hCG assay. Receiver operating characteristics (ROC) curves were constructed to determine hCG cutoff levels and sensitivity between patients cured on single-agent chemotherapy (control group) and patients requiring change to combination chemotherapy (study group). Receiver operating characteristics analysis identified an hCG cutoff value of 737 IU l^−1^ that enabled us to predict the subsequent development of single-agent chemotherapy resistance in 52% of patients before their fourth MTX course at 97.5% specificity. This would have enabled an earlier switch to combination chemotherapy reducing the MTX exposure by an average of 2.5 courses. The present findings confirm that serum hCG cutoff levels predict resistance to single-agent therapy earlier than traditional methods. Change to combination chemotherapy should be considered for patients whose serum hCG levels exceed these hCG cutoff values. For patients not exceeding the hCG cutoff levels, static or rising hCG levels should still be included in the criteria for change of chemotherapy.

Human chorionic gonadotropin (hCG) is a sensitive marker in monitoring trophoblastic activity in gestational trophoblastic disease (GTD; Delfs, 1957). Persistent trophoblastic disease (PTD) is a clinical diagnosis, based on static or rising hCG levels following uterine evacuation of hydatidiform mole. The International Federation of Obstetrics and Gynecology (FIGO) defines PTD as static hCG levels in four consecutive blood samples, a rising hCG concentration in three consecutive samples or persistence of elevated hCG levels for more than 6 months after evacuation (FIGO Oncology Committee, 2002). Persistent trophoblastic disease occurs most commonly after complete hydatidiform moles (15%), although partial hydatidiform moles may also transform into malignant GTD (0.5%; Seckl *et al*, 2000; Wolfberg *et al*, 2004). Depending on their prognostic risk category, patients are generally treated with first-line single-agent chemotherapy (methotrexate (MTX) or actinomycin D) in case of low-risk disease or first-line EMA/CO combination chemotherapy (etoposide, MTX, actinomycin D, cyclophosphamide and oncovin) in case of high-risk PTD (FIGO Oncology Committee, 2002). Furthermore, 17–36% of patients initially treated with low-risk MTX require a change of chemotherapy due to MTX resistance. To date, the most widely used marker for resistance to single-agent chemotherapy is a plateau or rise in serum hCG levels, which can obviously occur after several courses of treatment (Dobson *et al*, 2000; Garrett *et al*, 2002; McNeish *et al*, 2002; Matsui *et al*, 2005). Consequently, earlier detection of patients likely to develop resistance is desirable, so that more appropriate therapy can be given to cure the disease rapidly.

Recent work from Van Trommel *et al* (2006) has identified serum hCG cutoff points, that allowed early prediction of MTX resistance. This study was based on hCG levels of 79 patients cured by first-line MTX. At 97.5% specificity, 50% of low-risk PTD patients not responding to MTX (*n*=29) could be identified just before the start of the fourth MTX course. Previously, Rotmensch *et al* (1988) designed a 90th percentile log-exponential regression curve with hCG levels of 19 patients successfully treated with MTX. Other investigators suggested hCG cutoff points determined by ROC curves for identifying MTX refractory patients based on hCG regression in MTX responsive patients (Shigematsu *et al*, 2003).

Prolonged duration of chemotherapy due to belated identification of single-agent refractory disease will likely increase stress levels and adversely affect quality of life because the total duration of therapy is increased. Although early detection of patients unresponsive to single-agent therapy is favourable, hCG cutoff levels to identify resistant disease should be stringent, as starting unnecessary high-risk chemotherapy might elicit severe toxicities and advance menopause (Rustin *et al*, 1996; Bower *et al*, 1998).

Further validation of the study performed by Van Trommel *et al* (2006) on serum hCG cutoff levels as a predictor for resistance to single-agent chemotherapy (developmental study) is required before clinical implementation. Therefore, the purpose of this study is to validate and extend the findings obtained from the Dutch developmental study on prediction of the need for combination chemotherapy in a larger external British patient cohort, in a different clinical setting, with serum hCG values obtained using a different hCG radioimmunoassay (RIA) in an independent laboratory.

## Materials and methods

### Study population and setting

**Figure 1:**
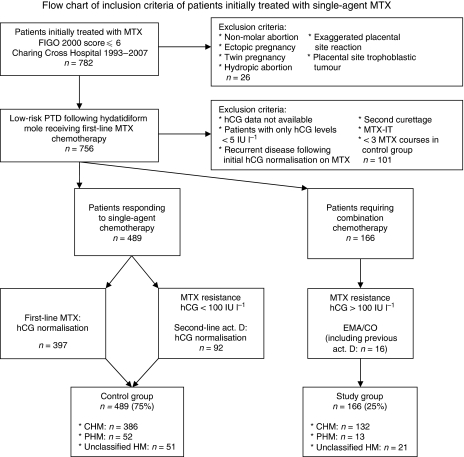
Flow chart of inclusion criteria of patients initially treated with single-agent MTX. MTX=methotrexate, FIGO=International Federation for Obstetrics and Gynecology, PTD=persistent trophoblastic disease, hCG=human chorionic gonadotropin, act. D=actinomycin D, EMA/CO=etoposide, MTX, actinomycin D, cyclophosphamide and oncovin, CHM=complete hydatidiform mole, PHM=partial hydatidiform mole, MTX-IT= intrathecal MTX.

Charing Cross Hospital, London, is one of the two centres in the United Kingdom for treatment of GTD. Between August 1993 and October 2007, 782 patients were treated with first-line MTX chemotherapy for low-risk PTD at Charing Cross Hospital. Exclusion criteria for further analysis are shown in Figure 1. A total of 655 patients with low-risk PTD, initially treated with single-agent chemotherapy, were included retrospectively in this study. Patients with an FIGO 2000 prognostic score ⩽6 (FIGO Oncology Committee, 2002) were instituted on first-line single-agent MTX chemotherapy with folinic acid rescue (50 mg intramuscular MTX on days 1, 3, 5 and 7 with folinic acid 30 mg orally on days 2, 4, 6 and 8, repeated every 2 weeks). Single-agent chemotherapy drug resistance was defined as two static or rising hCG levels (study end point). In the United Kingdom, for patients resistant to MTX, treatment was converted to second-line chemotherapy according to their hCG level, which is known to be associated with the amount of remaining trophoblastic tissue. Patients with MTX refractory disease are treated with second-line single-agent actinomycin D (hCG⩽100 IU l^−1^) or second-line combination chemotherapy (EMA/CO) in case of hCG levels >100 IU l^−1^ at the time of development of MTX resistance. A more reserved attitude towards treatment with combination chemotherapy has been introduced recently (hCG >100 IU l^−1^ recently became >300 IU l^−1^ for treatment with combination chemotherapy). This may increase the number of patients cured on single-agent chemotherapy. Actinomycin D (0.5 mg on days 1–5, repeated every 2 weeks) has shown to be a successful single-agent therapy for MTX resistant PTD, and did not compromise long-term outcome in patients subsequently requiring third-line combination chemotherapy (McNeish *et al*, 2002). Following normalisation of serum hCG levels (<5 IU l^−1^), treatment was continued for another 6 weeks (three consolidation courses), where after lifelong follow-up of hCG levels proceeded. Low-risk patients with pulmonary metastasis on chest X-ray received central nervous system prophylaxis with intrathecal MTX (MTX-IT) in addition to the first-line systemic MTX for possible occult cerebral metastases. Those patients were excluded from further analysis, as the additional effect of intrathecal MTX on systemic hCG levels is unknown.

### Immunoassays

In the United Kingdom, a non-commercial one-site in-house competitive hCG RIA using a rabbit polyclonal antibody is utilised for monitoring GTD. The antibody used in this RIA is directed to an epitope on the *β*-subunit and detects all known forms of *β*-hCG including free *β*, intact hCG and *β*-core fragment with a comparable cross-reactivity for the different forms of serum hCG. Furthermore, the antibody is not affected by nicked hCG and cross-reacts less than 0.25% with luteinising hormone of pituitary origin. The analytical sensitivity of the assay is 1 IU l^−1^. Human chorionic gonadotropin levels less than 5 IU l^−1^ are considered normal (Cole and Sutton, 2004).

### Statistical methods

Statistical analyses were performed using the SPSS statistical software package for Microsoft Windows (version 14.0, SPSS Inc, Chicago, IL, USA). Serum hCG levels were sorted within each MTX chemotherapy course number and data were excluded for further analysis from the moment therapy other than MTX started (hysterectomy, second curettage and second-line chemotherapy). Normalities of distributions were explored by means of the Kolmogorov–Smirnov test. Individual serum hCG levels were not normally distributed, neither with transformations. Therefore, we performed a non-parametrical analysis by means of ROC curves to determine hCG cutoff levels (decision threshold) for prediction of the need for combination chemotherapy (preceding each MTX course) at 99% specificity. Receiver operating characteristics curves were obtained from individual serum hCG levels grouped by MTX course to determine hCG cutoff levels (at 97.5 and 99% specificity), sensitivity (ie, percentage of patients identified) and area under curve (AUC). As sensitivity for prediction of the need for combination chemotherapy was dependent on the specificity level chosen and the AUC was not, we considered sensitivity to be the most important outcome measure. To make the results more accessible, we converted the absolute hCG levels given in IU l^−1^ also in percent rates of hCG serum levels. The conversion is based on 100%=23667 IU l^−1^, that is, the median (p50) hCG level of the study group before the first MTX course. Differences in numerical parametrical data were calculated by means of the two-tailed Student's *t*-test. Non-parametrical data were assessed by the use of the two-tailed Mann–Whitney *U*-test. *P*-values less than 0.05 were considered statistically significant. In case of patients <30 in numbers, data were censored.

## Results

A total of 782 patients received single-agent chemotherapy for low-risk PTD. Six hundred and fifty-five patients with low-risk persistent disease treated with single-agent chemotherapy between August 1993 and October 2007 fulfilled the inclusion criteria shown in Figure 1. Four hundred and eighty-nine patients (75%, control group) attained remission of trophoblastic disease on single-agent chemotherapy alone and 166 patients (25%, study group) required change to a combination chemotherapy regimen due to single-agent therapy resistance. Among the 489 patients who achieved remission on single-agent chemotherapy, 92 patients (18%) achieved remission on second-line single-agent actinomycin D following MTX chemotherapy. One hundred and sixty-six patients required combination chemotherapy, including 16 patients (10%) who previously received second-line actinomycin D following MTX chemotherapy.

Table 1 describes clinical and therapy-related characteristics of patients initially treated with single-agent chemotherapy. In the case of 48 and 19 patients in the control and study group, respectively, evacuation dates were not available in the registry's database. Those patients were not eligible for the analysis of maternal age and start date of single-agent chemotherapy. Maternal ages at the time of evacuation did not differ comparing the control group with the study group. However, start date of MTX chemotherapy was significantly further from evacuation in the control group when compared with the study group (median 10 *vs* 7 weeks, *P*<0.0001). Overall, for 95% of patients, the first-line MTX was administered within 26 weeks from evacuation. As expected, prechemotherapy hCG levels were statistically lower in the control group than in the study group (median 4614 IU l^−1^=19% *vs* 23667 IU l^−1^=100%, *P*<0.0001). Individuals from the control group received significantly more MTX courses than those among the study group (median, six *vs* four courses, *P*<0.0001). The number of patients that received second-line actinomycin D in the study group (*n*=16, median, two courses) was too low to be compared with the patients that received second-line actinomycin D in the control group (*n*=92, median five courses). The median number of courses EMA/CO combination chemotherapy for patients in the study group was five.

**Figure 2:**
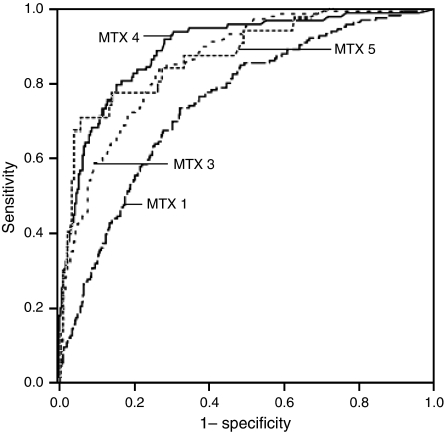
Receiver operating characteristics curves of serum hCG levels in patients requiring combination chemotherapy (study group) compared with hCG levels among the patients cured on single-agent chemotherapy (control group) before the first, third, fourth and fifth MTX course (MTX 1, 3, 4 and 5). hCG=human chorionic gonadotropin.

Table 2 shows the non-parametric assessment of hCG cutoff levels by means of ROC curves (represented in Figure 2) derived from serum hCG levels from patients who gained cure on single-agent therapy compared with those who required combination chemotherapy, sorted per MTX course number. We evaluated hCG cutoff levels and sensitivity both at the 97.5 and 99% specificity level. Before the start of MTX chemotherapy, 19% of patients eventually requiring combination chemotherapy could have been identified by means of hCG cutoff levels (hCG cutoff: 57315 IU l^−1^=242%), whereas before the fourth course, sensitivity increased to 52% (hCG cutoff: 737 IU l^−1^=3%) at 97.5% specificity. When using the more strict 99% specificity level to prevent unnecessary change to combination chemotherapy, sensitivity decreased, whereas hCG cutoff levels increased. Before the start of the first MTX course (hCG cutoff: 121664 IU l^−1^=514%), 9% of all MTX-resistant patients could be predicted at 99% specificity. This percentage increased to 26% before the fourth course (hCG cutoff: 1580 IU l^−1^=7%).

Moreover, for single-agent chemotherapy-resistant PTD with hCG levels exceeding one or more hCG cutoff levels before the first, second, third, fourth or fifth MTX course at 97.5% specificity, the number of MTX chemotherapy courses could have been reduced, in retrospect, by an average of 2.5 MTX courses (range, 1–9 courses). In case the hCG cutoff levels at 99% specificity would have been applied, an average of 2.1 MTX courses (range, 1–6) could have been saved. These data are not shown in Table 2.

**Figure 3:**
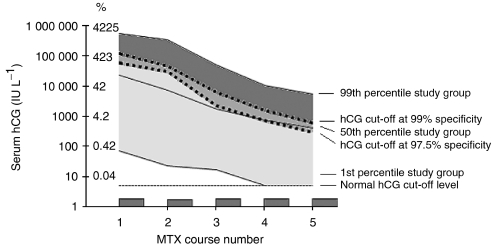
Serum hCG regression in patients requiring combination chemotherapy (study group) compared with serum hCG cutoff levels at 99 and 97.5% specificity based on ROC analysis of hCG levels among the study *vs* the control group. hCG=human chorionic gonadotropin, MTX=methotrexate.

Figure 3 illustrates hCG cutoff levels derived from ROC analysis based on serum hCG regression in the study group, when compared with the hCG levels among the control group. For patients with hCG levels in the dark grey area, the need for combination chemotherapy could be predicted by means of exceeding the hCG cutoff levels when specificity was set at 99%. Patients with hCG levels in the medium grey area could additionally be identified in case hCG cutoff levels at 97.5% specificity would have been applied. Patients with hCG levels in the light grey area could not be predicted by exceeding of the hCG cutoff levels at 97.5 or 99% specificity. In addition to absolute hCG levels, the Y-axis shows the hCG levels converted to percent rates, based on 100%=23667 IU l^−1^, that is, the median of serum hCG levels among the study group before the first MTX course.

## Discussion

The purpose of this study was to validate the Dutch developmental study on prediction of the need for combination chemotherapy by means of hCG cutoff levels performed by Van Trommel *et al* (2006) using a much larger retrospective PTD patient data set from a different organisation with a distinct hCG assay. A six times larger patient number in the present validation study enabled us to refine the model for determining hCG cutoff levels as developed earlier by Van Trommel *et al* (2006). First, to reduce the false-positive diagnosis of single-agent chemotherapy resistance, we calculated the specificity level at 99% in addition to the 97.5% specificity level. A further adjustment of the calculations performed by Van Trommel *et al* (2006) was the removal of all hCG levels from the moment of hCG normalisation in the control group. This is because, in the prospective setting, patients who achieve normalisation are not considered for further treatment. Consequently, hCG cutoff levels increased, and there was a slight decrease in the calculated predictive value for MTX resistance relative to that of a calculation including all normal hCG levels. The last refinement in the model for determining hCG cutoff levels for prediction of MTX resistance is that we have calculated those hCG cutoff levels by non-parametric analysis only, as the present serum hCG data were not normally distributed, whereas Van Trommel *et al* (2006) used both parametric (log-transformed hCG regression lines) and non-parametric assessment (derived from ROC analysis).

One-fourth of all patients initially treated for low-risk PTD with single-agent chemotherapy subsequently required combination chemotherapy. For those patients requiring combination chemotherapy, serum hCG cutoff levels could predict 26% of single-agent chemotherapy-resistant patients before the fourth MTX course at 99% specificity. Interestingly, a 1.5% decrease in specificity (from 99 to 97.5%) gives rise to a two times higher sensitivity (from 26 to 52%), although earlier identification of patients requiring combination chemotherapy at the expense of a decrease in specificity (ie, false-positive diagnosis of single-agent chemotherapy refractory disease) is unfavourable. For MTX refractory patients with one or more serum hCG levels exceeding the hCG cutoff levels before the first, second, third, fourth or fifth MTX course at 97.5% specificity, the number of MTX chemotherapy courses could have been reduced, in retrospect, by an average of 2.5 courses (range, 1–9). Comparison of absolute hCG cutoff levels of this study with hCG cutoff levels derived from the developmental study of Van Trommel *et al* (2006) is not applicable, as a different RIA was used, with different sensitivity, specificity and cross-reactivity. Confirmatory data using a distinct much larger patient cohort with another RIA of course further strengthens our model. Using hCG cutoff levels at 97.5% specificity, we found that 52% of patients resistant to single-agent chemotherapy could be identified before the fourth MTX course, which is comparable to 50% of MTX-resistant patients predictable in the Dutch developmental study. The percentage of MTX-resistant patients identified by means of hCG cutoff levels might be influenced by the low number of MTX-resistant patients in the Dutch cohort (*n*=29) and a 10 times higher cross-reactivity for *β*-hCG in the Dutch assay, which may overestimate the difference in hCG levels between MTX responsive and resistant patients and the method of assessment of hCG cutoff levels. Also, in this study, the control group consisted of patients cured on single-agent chemotherapy (including MTX and actinomycin D), whereas the control group in the developmental study consisted of patients cured on MTX solely. The serum hCG levels among the subgroup who received MTX-only were comparable to those observed in the total single-agent chemotherapy group. The higher median number of MTX courses in the Dutch group than in the Charing Cross group of patients eventually requiring combination chemotherapy (seven *vs* four courses of first-line MTX) suggest a more proactive approach towards change to combination therapy in the latter.

Prospective testing of a parameter on an independent set of patients in a multicentre setting is the ultimate test a parameter has to go through before reaching the level of evidence (LOE) type I according to the Tumor Marker Utility Grading Scale as introduced by Hayes *et al* (1996). Because of the retrospective nature of our external validation study, we considered the LOE to be III out of V. Unfortunately, a prospective study is impracticable, as low-risk PTD is a rare condition. Confounding factors due to the retrospective nature of this study were for instance different criteria for low-risk disease in force at the time. However, MTX treatment protocols and criteria for MTX resistance did not diverge within this study, enabled by centralised treatment of PTD at one specialized centre. Although LOE type I is not reached, we consider our parameter suitable for the application in the routine clinical practice.

In this study, 39% of patients initially classified as low-risk PTD, developed resistance to MTX, an apparent increase compared with resistance rates reported by the Charing Cross Hospital before (1989: 20%, 2002: 31%; Bagshawe *et al*, 1989; McNeish *et al*, 2002). In 1989, patients were classified as low-risk PTD with a Charing Cross score from 0 to 5 (⩽8 corresponds to an FIGO score ⩽6) (Bagshawe *et al*, 1989). The subsequent inclusion of the former medium risk group of patients scoring 6–8 on the Charing Cross Score likely accounts for the increased MTX resistance was seen. Indeed, the report from 2002 included several patients who were classified as low-risk PTD with a Charing Cross score between 0 and 8 (McNeish *et al*, 2002). This study incorporated more patients who were classified as low-risk PTD by the revised classification, resulting in a higher proportion of higher prognostic risk scores, and thus an increased MTX resistance rate.

In conclusion, we confirm the earlier observation by Van Trommel *et al* (2006) that hCG cutoff levels are useful for the prediction of resistance to single-agent chemotherapy. Change to combination chemotherapy should be considered for patients whose serum hCG levels exceed the cutoff levels. For patients not exceeding the hCG cutoff levels, static or rising hCG levels should still be included in the criteria for change of chemotherapeutic regimen following single-agent chemotherapy. Our model of prediction of the need for combination chemotherapy proved to be valid on an independent group of patients with hCG values obtained using a different hCG assay.

## Figures and Tables

**Table 1:** Clinical and therapeutic features of patients initially treated with MTX chemotherapy

	**Control group *n*=489**	**Study group *n*=166**	***P*-value**
*Maternal age (years)*			
Median (range)	30 (18–46)	31 (21–45)	NS^a^
			
*Start MTX (weeks after evacuation)*			
Median (range)	10 (4–27)	7 (3–31)	<0.0001^a^
			
*Prechemotherapy hCG (IU l*^*−1*^)			
Median (range)	4614 (50–5.8^*^10^4^)	23667 (638–1.8^*^10^5^)	<0.0001^a^
			
*Number of courses MTX*			
Median (range)	6 (3–10)	4 (2–6)	<0.0001^a^
			
*Number of courses actinomycin D*			
Median (range)	5 (1–7)	2 (1–5)	NA
			
*Number of courses EMA/CO*			
Median (range)	NA	5 (2–11)	NA

EMA/CO=etoposide, MTX, actinomycin D, cyclophosphamide, oncovin; hCG=human chorionic gonadotropin; MTX=methotrexate; NS=not significant; NA=not applicable.

Range=5th to 95th percentile.

a Mann–Whitney *U*-test.

**Table 2:** AUC and sensitivity at 97.5 and 99% specificity based on ROC curves of serum hCG levels before the start of MTX chemotherapy (MTX 1) and before the third, fourth and fifth MTX course (MTX 3, 4 and 5)

	**MTX 1**	**MTX 3**	**MTX 4**	**MTX 5**
*Control group (n)*	489	466	285	169
hCG cutoff (IU l^−1^) at 97.5% specificity	57 315	2269	737	304
hCG cutoff (IU l^−1^) at 99% specificity	121 664	6433	1580	600
				
*Study group (n)*	166	148	98	30
AUC (95% CI)	0.75 (0.71–0.79)	0.86 (0.83–0.93)	0.89 (0.86–0.93)	0.87 (0.80–0.95)
Sensitivity at 97.5% specificity	19%	43%	52%	67%
Sensitivity at 99% specificity	9%	20%	26%	30%

AUC=area under curve; CI=confidence interval; hCG=human chorionic gonadotropin; ROC=receiver operator characteristics.
